# Good performance of the criteria of American College of Medical Genetics and Genomics/Association for Molecular Pathology in prediction of pathogenicity of genetic variants causing thoracic aortic aneurysms and dissections

**DOI:** 10.1186/s12967-022-03251-8

**Published:** 2022-01-25

**Authors:** Joanna Kinga Ponińska, Zofia Teresa Bilińska, Grażyna Truszkowska, Ewa Michalak, Anna Podgórska, Małgorzata Stępień-Wojno, Przemysław Chmielewski, Anna Lutyńska, Rafał Płoski

**Affiliations:** 1 Department of Medical Biology, National Institute of Cardiology, Alpejska 42, Warsaw, Poland; 2 Unit for Screening Studies in Inherited Cardiovascular Diseases, National Institute of Cardiology, Alpejska 42, Warsaw, Poland; 3 Department of Medical Genetics, Centre of Biostructure, Medical University of Warsaw, Pawińskiego 3c, Warsaw, Poland

**Keywords:** Thoracic aortic aneurysm and dissections, Next-generation sequencing, Genetic variant classification, Recommendations of American College of Medical Genetics and Genomics/Association for Molecular for Molecular Pathology

## Abstract

**Background:**

The identification of pathogenic variant in patients with thoracic aortic aneurysms and dissections (TAAD) was previously found to be a significant indicator pointing to earlier need for surgical intervention. In order to evaluate available methods for classifying identified genetic variants we have compared the event-free survival in a cohort of TAAD patients classified as genotype-positive versus genotype-negative by the American College of Medical Genetics and Genomics and the Association for Molecular Pathology (ACMG-AMP) criteria or by ClinVar database.

**Methods:**

We analyzed previously unreported cohort of 132 patients tested in the routine clinical setting for genetic variants in a custom panel of 30 genes associated with TAAD or the TruSight Cardio commercial panel of 174 genes associated with cardiac disease. The identified variants were classified using VarSome platform. Kaplan–Meier survival curves were constructed to compare the event-free survival between probands defined as ‘genotype-positive’ and ‘genotype-negative’ using different classifications in order to compare their performance.

**Results:**

Out of 107 rare variants found, 12 were classified as pathogenic/likely pathogenic by ClinVar, 38 were predicted to be pathogenic/likely pathogenic by ACMG. Variant pathogenicity as assessed by ACMG criteria was a strong predictor of event free survival (event free survival at 50 years 83% vs. 50%, for genotype positive patients vs. reference, respectively, p  = 0.00096). The performance of ACMG criteria was similar to that of ClinVar (event free survival at 50 years 87% vs. 50%, for genotype positive patients vs. reference, respectively p  = 0.023) but independent from it as shown by analysing variants with no ClinVar record (event free survival at 50 years 80% vs. 50%, p  = 0.0039). Variants classified as VUS by ACMG criteria or ClinVar did not affect event-free survival. TAAD specific custom gene panel performed similar to the larger universal cardiac panel.

**Conclusions:**

In our cohort of unrelated TAAD patients ACMG classification tool available at VarSome was useful in assessing pathogenicity of novel genetic variants. Gene panel containing the established genes associated with the highest risk of hereditary TAAD (*ACTA1*, *COL3A1*, *FBN1*, *MYH11*, *SMAD3*, *TGFB2*, *TGFBR1*, *TGFBR2*, *MYLK*) was sufficient to identify prevailing majority of variants most likely to be causative of the disease.

**Supplementary Information:**

The online version contains supplementary material available at 10.1186/s12967-022-03251-8.

## Background

There is a growing interest in better recognition of genetic factors leading to thoracic aortic aneurysms and dissections (TAAD) [1, 2]. Diagnosis of TAAD leads to important clinical decisions. Although aortic size remains the main criterion for prophylactic surgical intervention, recent evidence indicates that aortic dissection may occur in nondilated or mildly dilated aorta, so aortic size loses its predictive ability [3–5]. Early diagnosis and subsequent prophylactic management prior to dissection significantly improves survival [6].

Previously, we described 51 TAAD patients studied with the use of whole exome sequencing (WES) or panel analysis (TruSight One, Illumina). We have reported a significant difference in event-free survival between ‘genotype-positive’ group consisting of patients with variants considered as pathogenic/likely pathogenic (P/LP) on account of either literature reports, protein disruption, de novo occurrence, segregation analysis or strong pathogenicity predictions as compared to ‘genotype-negative’ patients [7].

In the present work we analyzed an independent cohort of 132 patients tested in the routine clinical setting for variants in 30 genes associated with TAAD or 174 genes included in TruSight Cardio panel (Illumina). Considering the recent progress in development of free online databases collecting knowledge on human genetic variation we aimed to evaluate the performance of variant classification based on recommendations of American College of Medical Genetics and Genomics and the Association for Molecular Pathology (ACMG-AMP) [8]. The evaluation was based on a comparison of the event-free survival in patients classified as genotype-positive vs. genotype-negative by the ACMG-AMP criteria or by the expert curated information from the ClinVar database.

## Methods

### Patients and consent

The study cohort was chosen from all index patients referred with the diagnosis of TAAD for clinical genetic testing from 2012 to 2019 to the Unit for Screening Studies in Inherited Cardiovascular Diseases, Institute of Cardiology and comprised 132 unrelated patients (all Caucasian). Patients with family history of aortic dissection aortic aneurysm, unexplained sudden death in first-degree relatives, early onset of aortic disease, suspected connective tissue disorders were included primarily. In all patients a three-to-four generation pedigree was drawn and the data on the presence of TAAD, and other diseases in the family were collected. Every effort was made to review medical data on deceased subjects to confirm familial form of TAAD. For patients suspected of Marfan syndrome (MFS) and their relatives revised Ghent criteria were used [9] and a detailed questionnaire was applied to define the involvement of other systems and organs. Systemic score for each patient was calculated with web calculator http://www.marfan.org/resources/professionals/marfandx. In addition, web questionnaires were used to assess systemic features of Loeys-Dietz syndrome http://www.loeysdietz.org/. With regard to cardiovascular system, all patients had Doppler echocardiographic study and CT scan of the entire aorta. In particular, we collected the following data: age at diagnosis of thoracic aortic aneurysm (TAA), the history of acute aortic dissection (AAD) or prophylactic thoracic aorta surgery. Indications for elective surgery relied on available guidelines and evolved with time. Recognition of aortic root dilatation was based on echocardiography with calculation of Z-score for aortic root using Web calculator http://www.marfan.org/resources/professionals/marfandx. Ascending aorta dimensions were normalized to body surface area. According to guidelines, patients were followed-up with serial examinations by two-dimensional echocardiography and/or CT scan of the aorta.

Aortic events were defined as either acute aortic dissection (AAD) or first elective aortic surgery. The result of genetic testing did not affect the timing of first surgery in any patients from the study cohort. Data concerning mitral valve included presence of mitral valve prolapse (MVP) and mitral regurgitation (MR) on echocardiography. Diagnosis for MVP was based upon published criteria [10]. Left ventricular noncompaction was diagnosed based on CMR study with the ratio of noncompacted to compacted myocardium greater than 2.3 during diastole on long-axis cine images [11].

Hypertrophic cardiomyopathy was defined as left ventricular hypertrophy in the absence of loading conditions, sufficient to account for the observed degree of hypertrophy, with a maximal left ventricular wall thickness  ≥ 15 mm in one or more myocardial segments [12].

Familial disease was defined as the presence of  > 1 patient with TAAD in the family. This study was approved by the Bioethics Committee in the Institute of Cardiology (Ref. No. 1407). All participants of the study gave an informed written consent including specific consent to genetic testing and permission to publish the results.

### Genetic testing

Custom-designed (SeqCap, Roche) panel consisting of 30 genes related to aorthopathies and connective tissue disorders (Table 1) was used for sequencing in 102 patients. A commercial panel (TruSight Cardio, Illumina) consisting of 174 genes associated with heritable cardiac disorders including 18 related to TAAD was used in 30 patients. Sequencing was performed on MiSeq Dx (Illumina).

**Table 1:** Analyzed genes

No.	Gene	OMIM #	Inheritance
1	***ACTA2***	102620	AD
2	*ADAMTS10*	608990	AR
3	***CBS***	613381	AR
4	*COL1A1*	120150	AD
5	***COL3A1***	120180	AD
6	***COL5A1***	120215	AD
7	*COL5A2*	120190	AD
8	***EFEMP2***	604633	AR
9	***ELN***	130160	AD
10	*FBLN5*	604580	AR
11	***FBN1***	134797	AD
12	***FBN2***	612570	AD
13	*FLNA*	300017	XL
14	*HCN4*	605206	AD
15	***LTBP2***	602091	AR
16	*MAT2A*	601468	AD
17	*MFAP5*	601103	AD
18	***MYH11***	160745	AD
19	***MYLK***	600922	AD
20	***NOTCH1***	190198	AD
21	*NUP43*	608141	AD
22	*PLOD1*	153454	AR
23	*PRKG1*	176894	AD
24	*SKI*	164780	AD
25	***SLC2A10***	606145	AR
26	***SMAD3***	603109	AD
27	***TGFB2***	190220	AD
28	***TGFB3***	190230	AD
29	***TGFBR1***	190181	AD
30	***TGFBR2***	190182	AD

Bold characters—genes present in both panels

*AD* autosomal dominant; *AR* autosomal recessive; *XL* X-linked

### Variants assessment

We analysed variants located in the coding or splicing regions within genes of interest, of frequency no greater than 0.001 for autosomal dominant and X-linked inheritance pattern or 0.01 for autosomal recessive in both gnomAD genomes and gnomAD exomes databases. Pathogenicity, including VUS status, was assessed using ClinVar database classification and according to guidelines of ACMG-AMP using ACMG Classification tool (version 9.1.2) provided by Varsome platform and described in detail: https://varsome.com/about/resources/acmg-implementation. Among other criteria, VarSome uses consensus of the following in silico pathogenicity predicting programmes with default settings: BayesDel_addAF, DANN, DEOGEN2, EIGEN, FATHMM-MKL, LIST-S2, M-CAP, MVP, MutationAssessor, MutationTaster, SIFT, PrimateAI while PhyloP100Way (conservation assessment) is used with cut offs -3.387, 3.858, 6.8, 7.2. The details of Varsome implementation of ACMG criteria are given at: https://varsome.com/about/resources/acmg-implementation/.

Variation was considered novel when absent from ClinVar and HGMD database (release 2020.3), and had no other literature reference according to VarSome database. Thus identified P/LP variants and variants of unknown significance (VUS) were confirmed by Sanger sequencing.

### Statistical analysis

Kaplan–Meier survival curves were constructed to compare the event-free survival between probands defined as ‘genotype-positive’ and ‘genotype-negative’ using different classification tools in order to compare their performance. Online tool available at https://astatsa.com/LogRankTest/ was used. Aortic events were defined as acute aortic dissection or first planned aortic surgery. For the purpose of constructing genotype positive and genotype negative groups variants with “conflicting interpretation of pathogenicity” status in ClinVar were classified according to prevailing interpretation and in absence of such the less categorical interpretation was chosen (e.g., likely benign over benign or VUS over likely pathogenic). Finally, ‘genotype-negative’ reference group was created consisting of patients with no rare variant found or with no other variant(s) then benign or likely benign (B/LB) by ClinVar classification. One patient who had a likely benign variant according to ClinVar classified as likely pathogenic by ACMG was removed from the reference group.

## Results

### Clinical findings

Table 2 shows summarized clinical characteristics of the study group. Mean age at the time of genetic inquest was 43.4 ± 11.3 years and 92 (69.7%) patients were male. Of the 132 patients, 82 had aortic events (62.2%), in 39 (29.5%) patients AAD was the first symptom of the disease at mean age of 43 years, and 43 (32.6%) had planned aortic surgery at mean age of 39 years as the first procedure. Eight patients required another surgical procedure during follow-up after the first operation for AAD, and three of them had subsequent procedure. Of the 43 patients who had a planned procedure only, 3 required another surgical treatment. AAD following any planned procedure occurred in 3 patients.

**Table 2:** Clinical characteristics of the study group, n  = 132

Age at the genetic inquest (years)	44.9 ± 14.2	
Male sex (n, %)	92	69.7%
AAD first event (n, %)	39	29.5%
Age at AAD (years)	43.4 ± 11.2	
AAD type: Stanford A/B	36/3	
Planned TAA first procedure (n, %)	43	32.6%
Age at planned TAA first procedure (years)	39.4 ± 14.2	
TAA with no criteria for surgery	50	37.9%
Age (years)	45.5 ± 16.0	
Dilation of the aortic root, z score ≥ 2	43	32.6%
Aortic root dimension (mm, z score)	44.6 ± 4.5	3.9 ± 1.3
Dilation of the ascending aorta normalized to BSA > 2	27	20.5%
Ascending aorta dimension (mm, normalized to BSA)	46.3 ± 3.7	2.4 ± 0.2
Dilation of both aortic root and ascending aorta	20	15.2%
Age at last clinical clinical examination (years)	47.3 ± 13.8	
Syndromic or familial disease		
MFS	24	18.2%
MFS-like	15	11.4%
Familial TAAD (n, %)	45	34.1%
AAD in first-degree relative	19	14.4%
Unexplained sudden death in first-degree relative	10	7.6%
Associated structural and functional cardiac abnormalities		
BAV	28	21.2%
Aortic regurgitation	35	26.5%
Aortic stenosis	8	6.1%
CoA and PDA	1	0.8%
MVP with MR (mild/moderate/severe)	9 (2/5/2)	6.8%
Left ventricular noncompaction	1	0.8%
Hypertrophic cardiomyopathy	2	1.5%
Other cardiovascular diseases		
Hypertension	73	55.3%
Coronary artery disease	20	15.2%
Stroke	8	6.1%
Peripheral artery aneurysms	6	4.5%
Co-existent abdominal aortic aneurysm	2	1.5%
Other diseases		
Obesity	20	15.2%
Diabetes mellitus	10	7.6%
Dyslipidemia	37	28.0%
Rheumatoid arthritis	2	1.5%

The remaining 50 (38.6%) patients were diagnosed with TAA at mean age of 45.5 years, however they did not meet criteria for surgical correction. Of the 50 patients with TAA, the majority (n  = 43) had aortic root dilatation with mean z score of 3.9 and dilation of both root and ascending aorta was found in 20/50 patients.

In 24 patients the diagnosis of MFS was made and in 15 patients with TAAD genetic examination was performed to confirm/exclude the diagnosis of MFS. In the whole group co-existing abnormalities included: bicuspid aortic valve—28 patients, coarctation of the aorta (CoA) and patent ductus arteriosus (PDA)—1 patient, MVP with MR (n  = 9), left ventricular noncompaction in one female patient with bradycardia and hypertrophic cardiomyopathy in two patients. Peripheral artery aneurysms were present in 6 (4.5%), history of stroke in 8 (6.1%), and coronary artery disease in 6 (11.7%) of patients. More than half of the whole study group 73 (55.3%) had hypertension. Familial disease was found in more than one third of TAAD patients (n  =  45, 34%). Of these, AAD in first degree relative was found in 19 patients (14.4%). In addition, a family history of unexplained sudden death was present in 10 (7.6%) subjects.

### Variant classification by ACMG and ClinVar criteria

Overall, 107 variants in 73 (55%) patients were identified. According to both ClinVar and ACMG, no compound heterozygotes or homozygotes for any pathogenic (P), likely pathogenic (LP), or VUS variant in any of the genes associated with recessive inheritance of TAAD were found. According to ClinVar, among genes associated with autosomal dominant or X-linked inheritance pattern 12 P/LP, 25 VUS, 23 B/LB and 47 variants with no record were found. According to the ClinVar classification, 12 patients (9.1%) had at least one P/LP variant, 10 patients (7.6%) had only B/LB variant(s) and 51 (38.6%) patients had only variant(s) of unknown status (either VUS or no record). Full list of identified variants is available in Additional file 1: Table S1.

Eleven out of 12 variants classified as P/LP by ClinVar were also predicted to be P/LP by ACMG criteria and one was classified as VUS, however another variant in the same patient was classified as P/LP by ACMG alone, so a total of 12 patients had variants classified by both ClinVar and ACMG as P/LP (in one patient classifications apply to 2 different variants). Most variants (60%) classified as VUS by ClinVar were predicted to be B/LB by ACMG; 32% were predicted as VUS and 8%—P/LP. Out of variants classified as B/LB by ClinVar alone one variant (4.3%) had conflicting (LP) prediction by ACMG. Among variants with no ClinVar record as much as 51.1% were predicted to be P/LP; 29.8% were predicted VUS and 19.1%—B/LB.

**Fig. 1:**
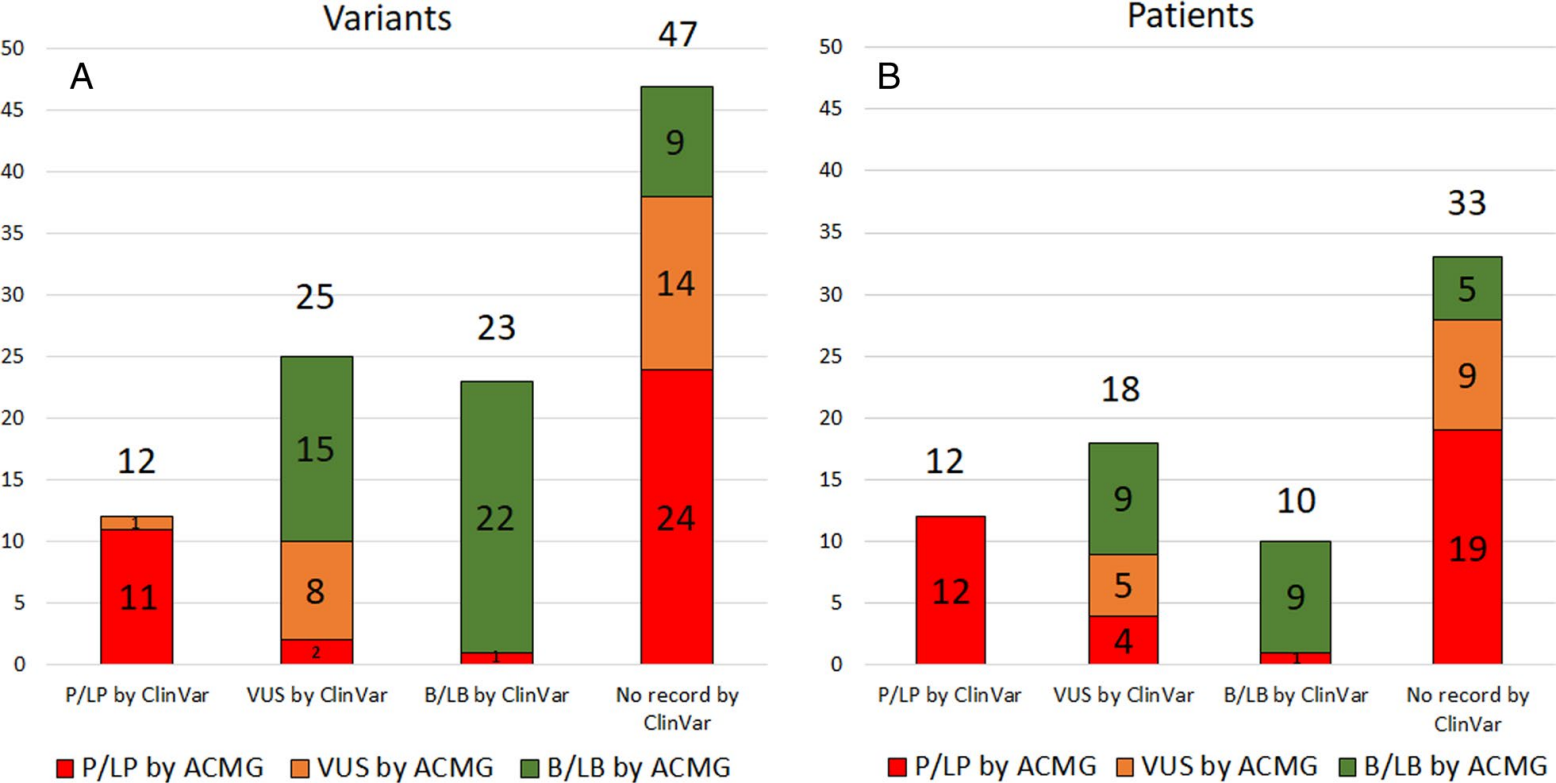
Distribution of ACMG predictions depending on the ClinVar classification **A** per variant, **B** per patient

Based on the ACMG classification 36 patients (27.3%) had at least one P/LP variant, 14 (10.6%)—at least one VUS with no P/LP variants and 23 (17.4%)—only B/LB variant(s). The distribution of ACMG predictions depending on the ClinVar classification is shown in Fig. 1.

### Variant pathogenicity as assessed by ACMG criteria is a strong predictor of event free survival

**Fig. 2:**
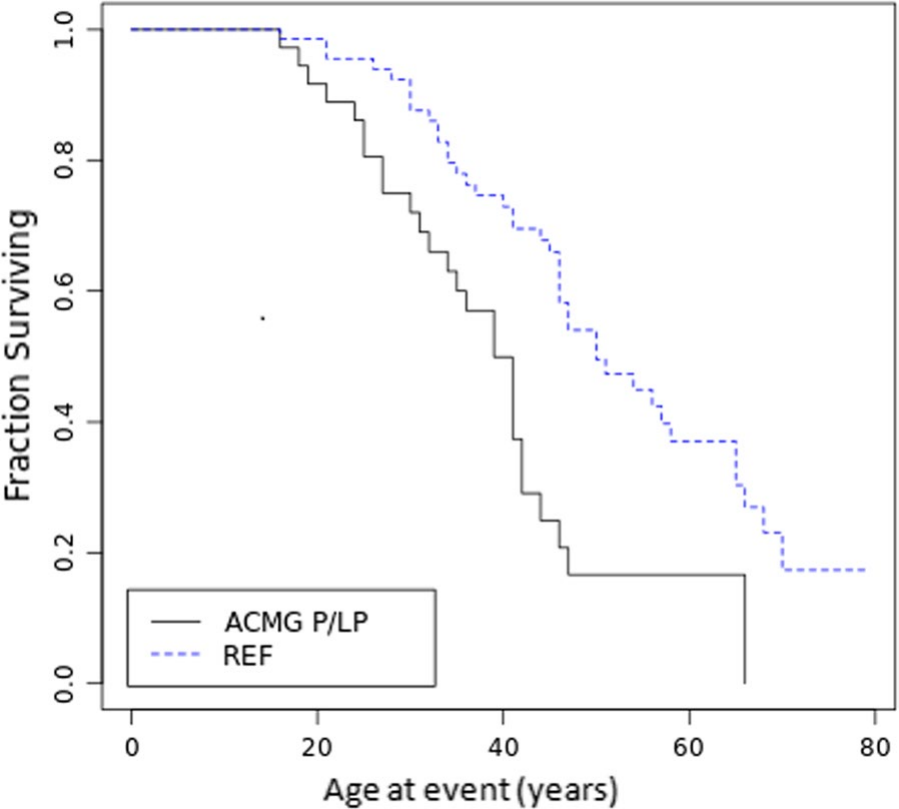
Kaplan–Meier analysis of event free survival in TAAD probands with variants classified as P/LP by ACMG (ClinVar included, AGMG P/LP) vs. those without any candidate variants found or variants classified as benign/likely benign by ClinVar and ACMG (REF, Log-Rank Chi- square 10.91, p  = 0.00096)

There was a highly significant difference in event-free survival when genotype positive group consisting of all patients with variants classified by ACMG as P/LP (including those listed in ClinVar) was compared to reference group of patients with no variant found or with variants classified as B/LB by both ClinVar and ACMG, p  = 0.00096. As can be seen, at 50 years of age 50% of patients from the reference group underwent surgery compared to 83% in genotype-positive group (Fig. 2).

We noted that the observed effect was found both among patients who suffered from AAD (p  = 0.02) and those who underwent prophylactic thoracic aorta surgery (p  = 0.006) with no apparent difference between the two groups (p  = 0.18, Additional file 1: Figure S1).

### The performance of ACMG criteria in predicating event free survival is independent from ClinVar information

**Fig. 3:**
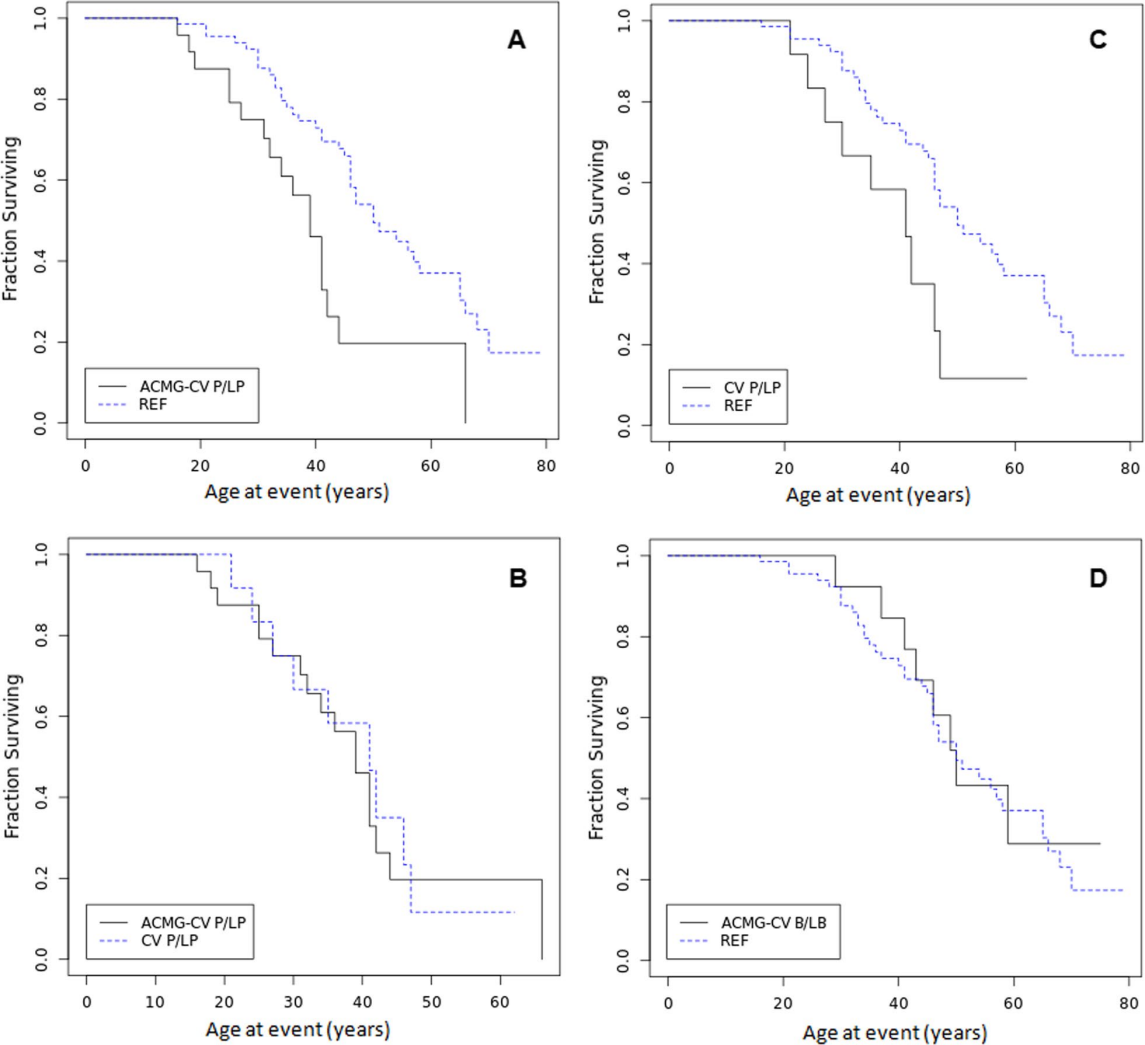
Kaplan–Meier analysis of event free survival in TAAD probands with variants classified as **A** P/LP by ACMG but not by ClinVar (ACMG-CV P/LP) vs. reference group (REF, Log-Rank Chi- square 8.32, p  = 0.0039); **B** P/LP pathogenic by both ClinVar and ACMG (CV P/LP) vs. P/LP pathogenic by ACMG alone (Log-Rank Chi- square 1.016, p  = 0.90); **C** P/LP by ClinVar alone vs. reference group (Log-Rank Chi-square 5.18, p  = 0.023), **D** B/LB by ACMG alone (ACMG-CV B/LB) vs. reference group (REF, Log-Rank Chi- square 0,028, p  = 0.87)

Since ClinVar status is included in ACMG criteria (PP5, BP6), the important question is to what extent prediction by ACMG criteria is independent from ClinVar data. In order to test this we analysed the data after dividing the ACMG positive group into those positive also according to Clinvar data (n  = 12) and those positive only by ACMG but not ClinVar (n  = 24). We found that the variants classified as P/LP by ACMG alone were associated with significantly shorter event-free survival compared to genotype-negative group, p  = 0.0039 (Fig. 3a). At 50 years of age 80% of patients from genotype-positive group underwent surgery compared to 50% on the reference group. Furthermore, there was no significant difference in event-free survival between 12 patients with P/LP variants according to both ClinVar and ACMG compared to 24 patients carrying variants predicted to be P/LP by ACMG criteria alone, p  = 0.90 (Fig. 3b).

As expected, the 12 variants classified by ClinVar as P/LP were associated with significantly shorter event-free survival compared to the reference group (patients with no rare variant or variant(s) classified by ClinVar as benign/likely benign (B/LB), p  = 0.023 (Fig. 3c). At 50 years of age 87% of patients from genotype-positive group underwent surgery.

There was also no significant difference in event-free survival between variants classified as B/LB by ACMG alone and the reference group, p  = 0.87 (Fig. 3d).

### Variants classified as VUS by either ACMG criteria or ClinVar do not affect event-free survival

**Fig. 4:**
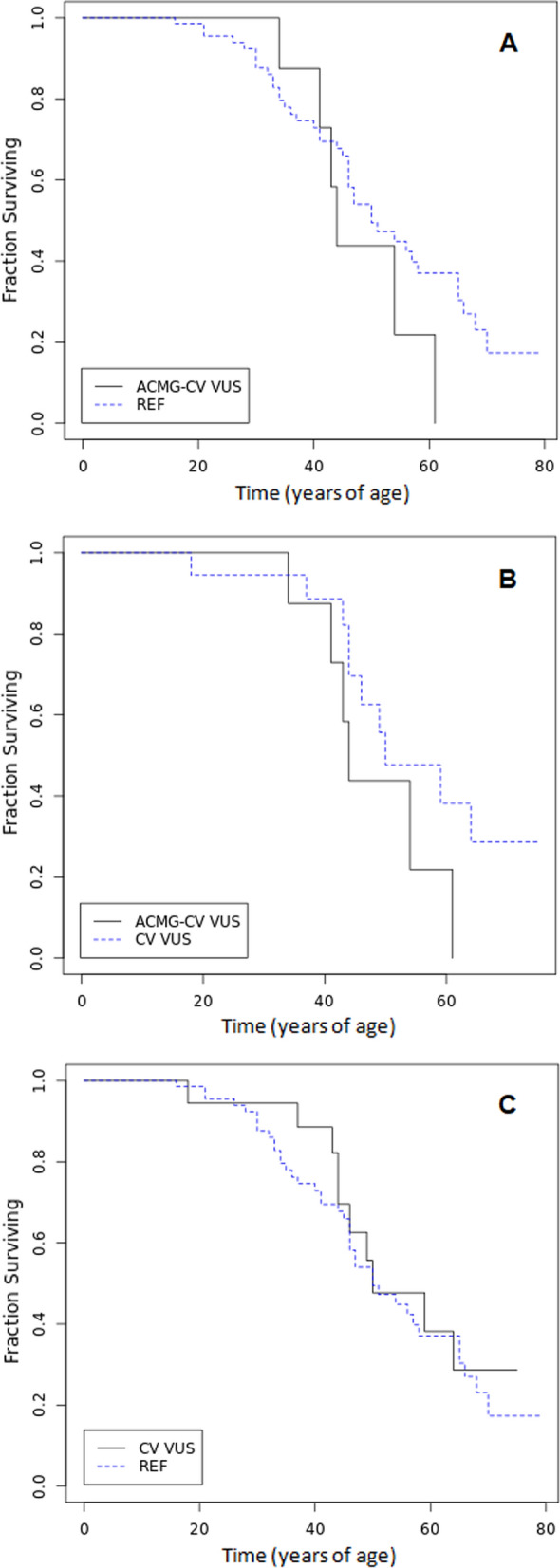
Kaplan–Meier analysis of event free survival in TAAD probands with variants classified as **A** VUS by ACMG alone (ACMG-CV VUS) vs. reference group (REF, Log-Rank Chi- square 0.86, p  = 0.35) (**B**); VUS by ACMG alone vs. VUS by ClinVar (CV VUS, Log-Rank Chi- square 1.80, p  = 0.18) **C** VUS by ClinVar vs. reference group (Log-Rank Chi- square 0.21, p  = 0.65)

In a similar way we analysed event-free survival in patients with variants classified as VUS but we found no significant difference either between those with variants classified as VUS by ACMG alone versus reference group (p  = 0.35, Fig. 4a), those with VUS by ACMG criteria alone compared to VUS by ClinVar (p  = 0.18, Fig. 4b) or those with VUS by ClinVar and reference group (p  = 0.65, Fig. 4c).

### Distribution of genes and variants types between P/LP, VUS and B/LB variants according to ACMG classification

Out of 38 variants classified as P/LP by ACMG, 28 (73,7%) were in *FBN1* gene, 2 variants (5.3%) in each of *SMAD3*, *TGFB2* and *TGFBR1*, 1 variant (2.6%) in each of the *COL3A1*, *HCN4*, *MYH11* and *MYLK* genes. 42% (16/38) variants were predicted to cause loss of function (LOF, nonsense, frameshift, splice site variants).

6 (26.1%) variants classified as VUS were in *FBN2*, 3 (13%) in *MYH11*, 2 (8.7%) in *ACTA2* and *MYLK*, 1 (4.3%) in each of *COL1A1*, *COL5A1*, *FLNA*, *HCN4*, *NOTCH1*, *NUP43*, *TGFB2*, *TGFBR2*, *ELN* and *COL3A1*. Twenty two percent (5/23) variants were predicted to cause LOF.

Most abundant among B/LB variants were variants in *COL5A1* gene—9 (19.6%); 8 (13.4%) in *NOTCH1*, 6 (13%) in *ELN*, 4 (8.7%) in *HCN4* and *MYH11*, 3 (6.5%) in *FLNA*, 2 (4.3%)—*TGFB3* and 1 (2.2%) in each of *ACTA2*, *COL1A1*, *FBN1* and *MFAP5*. Fifteen percent (7/46) variants were predicted to cause LOF.

We noted that among P/LP variants according to ACMG the LOF variants were associated with shorter event free survival (p  = 0.04, Additional file 1: Figure S2).

## Discussion

ClinVar being an expert knowledge-based database enables only the classification of variants with the existing evidence regarding observed health status. Novel variants and those lacking sufficient evidence add to the growing burden of VUS. In silico algorithms [13] add valuable supporting evidence regarding pathogenicity of given variant. The development of ACMG-AMP criteria which combine both lines of evidence with many levels of supporting information into an integrated prediction was a cornerstone in unifying interpretation of clinical significance of genetic variants. Since their initial development the criteria were evaluated by multiple laboratories, several areas for improvement were identified and there is an agreement on the need of further validation [14, 15]. In the present study we have tested and found good performance of an online tool available at varsome.com which allows classification based on ACMG-AMP criteria. Although the tool doesn’t cover all the criteria and is the subject to constant change in the algorithms implementing the ACMG-AMP criteria, it is free and convenient to use (https://varsome.com/about/resources/acmg-implementation/). The constantly improving functionalities of the AMG-AMP tool result in the growing number of more definitive predictions (B, LB, LP and P) and decreasing VUS classifications. Indeed, after application of ACMG-AMP criteria out of 72 variants having no ClinVar record or classified as VUS only 22 variants kept their VUS status (26 were classified as P/LP and 24 as B/LB). The goal of our study was to test the accuracy of these predictions using clinical data and ClinVar database as a reference. Similar to our previous findings [7]**,** patients carrying known pathogenic variants (P/LP by ClinVar), had AAD or were referred for elective aortic surgery significantly earlier than patients in whom no rare variants in genes of interest or only variants classified as B/LB by ClinVar were identified. Importantly, the genotype-positive group according to the ACMG verdict alone had similar (significant) difference in the event-free survival vs. the reference group as the group defined by ClinVar alone. Furthermore, there was no significant difference in event-free survival when we compared genotype-positive group according to ClinVar to patients carrying variants predicted to be P/LP by ACMG criteria alone. In the similar way, there was no significant difference between patients carrying only B/LB variants according to ACMG alone vs. the reference group. These findings indicate that ACMG predictions in regard to TAAD-susceptibility genes are of similar high clinical significance to information provided by ClinVar.

We also observed that among variants classified as pathogenic, those predicted to cause LOF were associated with higher severity then the remaining variants. Thus, variant type (LOF vs. non LOF) could provide additional clinically useful information. It is likely that in the future even better predictions will be possible based on knowledge collected for individual variants in sufficiently powered studies.

Interestingly, in our cohort of TAAD patients there was no significant difference in disease progression between patients carrying VUS by either ACMG or ClinVar vs. the reference group suggesting that variants lacking any sort of evidence for being P/LP are more likely to be B/LB, at least regarding being the cause of monogenic disease. However, it should be noted that this does not preclude their role as risk factors in multifactorial form of disorder. Interestingly, our data are consistent with the findings from the study of 1005 patients diagnosed with nonischemic dilated cardiomyopathy which analysed prognostic impact of disease-causing variants classified based on the ACMG-AMP criteria. The rate of major adverse cardiovascular events, end stage heart failure and malignant ventricular arrhythmia in 10-year follow-up was significantly higher in the genotype-positive group compared to the genotype-negative but showed no trend towards higher rate of these conditions among patients carrying VUS [16].

Our results also indicate that the TAAD specific panel performs similar to the larger universal cardiac panel. Out of 22 variants identified using TAAD-specific panel and predicted to be P/LP by ACMG and/or VarSome only one variant (in the *HCN4* gene) wouldn’t have been found if universal cardiac panel was used. This observation supports previous reports suggesting that use of smaller gene panels consisting of the most frequently mutated genes may be a cost-effective strategy as a first step of the genetic diagnosis for TAAD patients [17].

Furthermore, based on our analysis of TAAD patients, the P/LP variants we found by either ClinVar or ACMG resided mainly within the relatively few genes (*FBN1*, *SMAD3*, *TGFB2*, *TGFBR1*, *COL3A1*, *MYH11*, *MYLK*) generally agreed to be “definitively” associated with hereditary TAAD [2] and the highest risk of early fatal complications [18]. Patients with genetic defects in *TGFBR1*, *TGFBR2*, *SMAD3*, *TGFB2* (affecting TGF-β signalling pathway), *COL3A1* (extracellular matrix component), *FBN1* (TGF-β signalling affecting extracellular matrix component), *MYH11*, *ACTA2*, *MYLK* and *PRKG1* (structural components and modifiers of smooth muscle layer of the aorta) are at risk of dissection or rupture at aortic diameters less than 5.0 cm recommended for surgical intervention, and therefore sequencing is of key importance for decision-making regarding the time of prophylactic surgery [1].

## Conclusions

Gene panel containing the most established genes associated with the highest risk of early fatal complications in patients with hereditary TAAD (*ACTA1*,* COL3A1*,* FBN1*,* MYH11*,* SMAD3*,* TGFB2*,* TGFBR1*,* TGFBR2*,* MYLK*) was sufficient to identify prevailing majority of variants most likely to be causative of the disease.

ACMG classification tool available at varsome.com is useful in assessing pathogenicity of novel genetic variants in TAAD patients. Variant pathogenicity as assessed by ACMG criteria is a strong predictor of earlier AAD or need for surgical intervention. Hopefully, the progress in combining empiric data with the further development of bioinformatic tools will lead to the reduction in the number of VUS allowing even more reliable predictions that could be successfully used for clinical decisions.

## Supplementary Information

**Additional file 1: Table S1.** List of variants identified in TAAD patients. **Figure S1. **Kaplan–Meier analysis of event free survival in TAAD probands with variants classified as: **A** P/LP by ACMG (ACMG P/LP DISS) vs. reference group (REF DISS)—event = dissection prior to surgical intervention, Log-Rank Chi-square 5.23, p = 0.022), **B** P/LP by ACMG (ACMG P/LP PLAN) vs. reference group (REF PLAN)—event = planned prophylactic surgery, Log-Rank Chi-square 7.44, p = 0.0064, **C** P/LP by ACMG (ACMG P/LP DISS)—event = dissection prior to surgical intervention vs. P/LP by ACMG (ACMG P/LP PLAN) - event = planned prophylactic surgery, Log-Rank Chi-square 1.83, p = 0.18. **Figure S2. **Kaplan–Meier analysis of event free survival in TAAD probands with LOF variants classified as P/LP by ACMG (ACMG P/LP LOF) vs. missense and small in-frame deletions (ACMG P/LP MIS), Log-Rank Chi-square 4.16, p = 0.041.

## Data Availability

All the data not included in current article are available from the corresponding author on reasonable request.

## Abbreviations

AAD: Acute aortic dissection
ACMG-AMP: American College of Medical Genetics and Genomics and the Association for Molecular Pathology
BAV: Bicuspid aortic valve
B/LB: Benign/likely benign
BSA: Body surface area
CoA: Coarctation of the aorta
LDS: Loeys-Dietz syndrome
MFS: Marfan syndrome
MR: Mitral regurgitation
MVP: Valve prolapse
P/LP: Pathogenic/likely pathogenic
PDA: Patent ductus arteriosus
TAA: Thoracic aortic aneurysms
TAAD: Thoracic aortic aneurysms and dissections
VUS: Variants of unknown significance
WES: Whole exome sequencing
