# Supplementary material for: Good performance of the criteria of American College of Medical Genetics and Genomics/Association for Molecular Pathology in prediction of pathogenicity of genetic variants causing thoracic aortic aneurysms and dissections
Source: J Transl Med. 2022 Jan 25;20:42. doi: 10.1186/s12967-022-03251-8 (PMC8787943; doi:10.1186/s12967-022-03251-8)
Supplement: Supplementary file 1 — Additional file 1: Table S1. List of variants identified in TAAD patients. Figure S1. Kaplan–Meier analysis of event free survival in TAAD probands with variants classified as: A P/LP by ACMG (ACMG P/LP DISS) vs. reference group (REF DISS)—event = dissection prior to surgical intervention, Log-Rank Chi-square 5.23, p = 0.022), B P/LP by ACMG (ACMG P/LP PLAN) vs. reference group (REF PLAN)—event = planned prophylactic surgery, Log-Rank Chi-square 7.44, p = 0.0064, C P/LP by ACMG (ACMG P/LP DISS)—event = dissection prior to surgical intervention vs. P/LP by ACMG (ACMG P/LP PLAN) - event = planned prophylactic surgery, Log-Rank Chi-square 1.83, p = 0.18. Figure S2. Kaplan–Meier analysis of event free survival in TAAD probands with LOF variants classified as P/LP by ACMG (ACMG P/LP LOF) vs. missense and small in-frame deletions (ACMG P/LP MIS), Log-Rank Chi-square 4.16, p = 0.041. [file 12967_2022_3251_MOESM1_ESM.pdf]

Table S1. List of variants identified in TAAD patients.

No	Gene	hg38 position	Transcript position	Protein	Effect	ACMG Version: 9.1.2	ClinVar	ClinVar mean	Allele frefuency gnomad genomes	Allele frefuency gnomad_exomes
1	FBN1	15:048516225-G>A	NM_000138.4:c.1285C>T	NP_000129.3:p.Arg429Ter	stop_gained	Pathogenic	Pathogenic	P/LP	0	0
2	TGFBR1	9:099142664-G>A	NM_004612.4:c.934G>A	NP_004603.1:p.Gly312Ser	missense_variant	Pathogenic	Pathogenic/Likely pathogenic	P/LP	0.0000070	0.0000041
3	FBN1	15:048613054-C>T	NM_000138.4:c.203G>A	NP_000129.3:p.Cys68Tyr	missense_variant	Pathogenic	Pathogenic/Likely pathogenic	P/LP	0	0
	MYLK	3:123701493-A>G	NM_053025.3:c.2407T>C	NP_444253.3:p.Cys803Arg	missense_variant	Uncertain Significance	Uncertain significance	Uncertain significance	0.0000209	0.0000081
4	ELN	7:074057441-G>A	NM_000501.4:c.1358-199G>A		missense_variant	Benign	Uncertain significance	Uncertain significance	0.0003281	0.0001350
5	FBN1	15:048441799-A>G	NM_000138.4:c.6085T>C	NP_000129.3:p.Cys2029Arg	missense_variant	Likely Pathogenic	No record	No record	0	0
6	TGFB2	1:218363420-G>A	NM_003238.5:c.346+16373G>A		splice_region_variant	Uncertain Significance	No record	No record	0	0
7	MYH11	16:015740155-C>A	NM_002474.3:c.2893G>T	NP_002465.1:p.Ala965Ser	missense_variant	Benign	Likely benign/Uncertain significance	Uncertain significance	0.0003000	0.0003533
8	SMAD3	15:067165385-G>T	NM_005902.4:c.532+1G>T		splice_donor_variant	Pathogenic	No record	No record	0	0
9	FBN1	15:048508698-TCCATATCTG>T	NM_000138.4:c.1715-3_1720delCAGATATGG	NP_000129.3:p.Asp572_Cys576delinsGly	inframe_deletion	Likely Pathogenic	No record	No record	0	0
	ELN	7:074042986-G>A	NM_000501.4:c.328G>A	NP_000492.2:p.Ala110Thr	missense_variant	Benign	Benign(1);Likely benign(2);Uncertain significance(2)	B/LB	0.0004747	0.0004873
10	HCN4	15:073322861-G>A	NM_005477.3:c.3232C>T	NP_005468.1:p.Arg1078Cys	missense_variant	Uncertain Significance	Uncertain significance	Uncertain significance	0.0000209	0.0000185
11	COL5A1	9:134805027-G>A	NM_000093.4:c.3167G>A	NP_000084.3:p.Arg1056His	missense_variant	Uncertain Significance	Uncertain significance	Uncertain significance	0.0000070	0
12	NOTCH1	9:136506827-A>G	NM_017617.5:c.3790T>C	NP_060087.3:p.Cys1264Arg	missense_variant	Uncertain Significance	No record	No record	0	0
13	FBN1	15:048510171-T>C	NM_000138.4:c.1589-2A>G		splice_acceptor_variant	Pathogenic	No record	No record	0	0
14	FBN1	15:048508685-T>TA	NM_000138.4:c.1733dup	NP_000129.3:p.Arg579LysfsTer6	frameshift_variant	Likely Pathogenic	No record	No record	0	0
	MYH11	16:015719716-G>C	NM_002474.3:c.4954-3C>G		splice_region_variant	Uncertain Significance	No record	No record	0	0
15	FBN1	15:048460289-TTGACTTCCACA>T	NM_000138.4:c.5242_5252del	NP_000129.3:p.Cys1748LysfsTer15	frameshift_variant	Likely Pathogenic	No record	No record	0	0
16	FBN1	15:048644615-GC>G	NM_000138.4:c.156_157delGC	NP_000129.3:p.(p.Leu53Ter)	frameshift_variant	Likely Pathogenic	No record	No record	0	0
	FBN2	5:128335984-T>C	NM_001999.4:c.3724+4A>G		splice_region_variant	Uncertain Significance	No record	No record	0	0.0000081
17	NOTCH1	9:136508926-C>T	NM_017617.5:c.3115G>A	NP_060087.3:p.Gly1039Ser	missense_variant	Benign	Benign/Likely benign	B/LB	0	0.0003250
18	FBN1	15:048452676-C>T	NM_000138.4:c.5431G>A	NP_000129.3:p.Glu1811Lys	missense_variant	Likely Pathogenic	Likely pathogenic(3);Uncertain significance(1)	P/LP	0	0.0000041
19	TGFB2	1:218437462-C>T	NM_003238.5:c.1052C>T	NP_003229.1:p.Pro351Leu	missense_variant	Likely Pathogenic	Uncertain significance	Uncertain significance	0.0000140	0.0000163
	COL5A1	9:134699972-C>A	NM_000093.4:c.341C>A	NP_000084.3:p.Ala114Asp	missense_variant	Benign	Likely benign	B/LB	0.0007395	0.0005728
20	FBN1	15:048497380-A>T	NM_000138.4:c.2179T>A	NP_000129.3:p.Cys727Ser	missense_variant	Likely Pathogenic	No record	No record	0	0
21	FBN1	15:048600181-A>G	NM_000138.4:c.400T>C	NP_000129.3:p.Cys134Arg	missense_variant	Pathogenic	Pathogenic	P/LP	0	0
22	FBN1	15:048613009-C>G	NM_000138.4:c.247+1G>C		splice_donor_variant	Pathogenic	No record	No record	0	0
23	FBN1	15:048495519-C>A	NM_000138.4:c.2489G>T	NP_000129.3:p.Cys830Phe	missense_variant	Pathogenic	No record	No record	0	0
	COL5A2	2:189062866-G>A	NM_000393.5:c.1976C>T	NP_000384.2:p.Pro659Leu	missense_variant	Benign	Likely benign(3);Uncertain significance(2)	B/LB	0.0003638	0.0004843

	COL5A2	2:189179600-A>G	NM_000393.5:c.5T>C	NP_000384.2:p.Met2Thr	missense_variant	Likely Benign	Uncertain significance	Uncertain significance	0.0000070	0.0000437
	ELN	7:074042986-G>A	NM_000501.4:c.328G>A	NP_000492.2:p.Ala110Thr	missense_variant	Benign	Benign(1);Likely benign(2);Uncertain significance(2)	B/LB	0.0004747	0.0004873
	ELN	7:074065732-G>A	NM_000501.4:c.2032G>A	NP_000492.2:p.Gly678Ser	missense_variant	Benign	Uncertain significance	Uncertain significance	0.0000979	0.0000487
24	HCN4	15:073329702-CACGGGCGCCTGCCG>C	NM_005477.3:c.1447_1461del	NP_005468.1:p.Arg483_Val487del	inframe_deletion	Likely Pathogenic	No record	No record	0	0
25	FBN1	15:048537704-G>A	NM_000138.4:c.643C>T	NP_000129.3:p.Arg215Ter	stop_gained	Pathogenic	Pathogenic	P/LP	0	0
26	COL5A2	2:189043143-T>A	NM_000393.5:c.3471+8A>T		splice_region_variant	Benign	No record	No record	0.0006003	0.0005271
27	TGFB2	1:218346889-T>TC	NM_003238.5:c.194dup	NP_003229.1:p.Glu66GlyfsTer68	frameshift_variant	Likely Pathogenic	No record	No record	0	0
	COL1A1	17:050194815-A>G	NM_000088.3:c.1367T>C	NP_000079.2:p.Val456Ala	missense_variant	Uncertain Significance	No record	No record	0	0
28	TGFB3	14:075980601-G>A	NM_003239.4:c.293C>T	NP_003230.1:p.Ser98Leu	missense_variant	Benign	Benign(1);Likely benign(6);Uncertain significance(2)	B/LB	0.0009073	0.0007553
	MYLK	3:123708003-T>C	NM_053025.3:c.2141A>G	NP_444253.3:p.Glu714Gly	missense_variant	Likely Pathogenic	No record	No record	0.0000140	0.0000081
	TGFBR1	9:099144800-T>C	NM_004612.4:c.1042T>C	NP_004603.1:p.Cys348Arg	missense_variant	Likely Pathogenic	No record	No record	0	0
29	NOTCH1	9:136507350-C>T	NM_017617.5:c.3598G>A	NP_060087.3:p.Asp1200Asn	missense_variant	Benign	Uncertain significance	Uncertain significance	0.0000140	0.0000981
30	COL3A1	2:188993364-G>T	NM_000090.3:c.1054G>T	NP_000081.1:p.Glu352Ter	stop_gained	Pathogenic	No record	No record	0	0
	FBN2	5:128263641-G>C	NM_001999.4:c.7976C>G	NP_001990.2:p.Ser2659Cys	missense_variant	Uncertain Significance	No record	No record	0	0
31	NOTCH1	9:136523752-G>A	NM_017617.5:c.368C>T	NP_060087.3:p.Thr123Met	missense_variant	Benign	Benign/Likely benign	B/LB	0.0007396	0.0009510
	FBN2	5:128376802-T>C	NM_001999.4:c.1901A>G	NP_001990.2:p.Asn634Ser	missense_variant	Uncertain Significance	Uncertain significance	Uncertain significance	0	0
32	NOTCH1	9:136507379-T>C	NM_017617.5:c.3569A>G	NP_060087.3:p.His1190Arg	missense_variant	Likely Benign	Uncertain significance	Uncertain significance	0.0000140	0.0000083
33	COL5A1	9:134802904-C>T	NM_000093.4:c.3023C>T	NP_000084.3:p.Thr1008Met	missense_variant	Likely Benign	Likely benign(1);Uncertain significance(3)	Uncertain significance	0.0000907	0.0000786
34	FLNA	X:154352343-C>T	NM_001110556.2:c.6607G>A	NP_001104026.1:p.Val2203Ile	missense_variant	Likely Benign	Likely benign(1);Uncertain significance(1)	Uncertain significance	0	0.0000056
35	MYH11	16:015718337-C>T	NM_002474.3:c.5273G>A	NP_002465.1:p.Arg1758Gln	missense_variant	Benign	Likely benign(1);Uncertain significance(3)	Uncertain significance	0.0001954	0.0001566
36	TGFB3	14:075980601-G>A	NM_003239.4:c.293C>T	NP_003230.1:p.Ser98Leu	missense_variant	Benign	Benign(1);Likely benign(6);Uncertain significance(2)	B/LB	0.0009073	0.0007553
	MYH11	16:015715029-G>A	NM_002474.3:c.5666C>T	NP_002465.1:p.Ala1889Val	missense_variant	Benign	Uncertain significance	Uncertain significance	0.0000559	0.0000488
37	FBN1	15:048468057-TCTCCTCGAGGTCG>T	NM_000138.4:c.4615_4627del	NP_000129.3:p.Arg1539ThrfsTer38	frameshift_variant	Likely Pathogenic	No record	No record	0	0
38	MYH11	16:015721420-A>C	NM_002474.3:c.4578+2T>G		splice_donor_variant	Pathogenic	No record	No record	0	0
	ELN	7:074056290-AGTTGGAGGCATTCCTACTTACGGG>A	NM_000501.4:c.1178_1201del	NP_000492.2:p.Gly393_Gly400del	inframe_deletion	Benign	Likely benign(1);Uncertain significance(2)	Uncertain significance	0.0003428	0.0003655
	COL5A1	9:134824844-A>G	NM_000093.4:c.4943A>G	NP_000084.3:p.Asp1648Gly	missense_variant	Likely Benign	Uncertain significance	Uncertain significance	0.0000070	0.0000336
	NOTCH1	9:136497012-C>T	NM_017617.5:c.6727G>A	NP_060087.3:p.Gly2243Ser	missense_variant	Likely Benign	Uncertain significance	Uncertain significance	0.0000349	0.0000331
39	MYLK	3:123708769-G>A	NM_053025.3:c.2069C>T	NP_444253.3:p.Thr690Met	missense_variant	Uncertain Significance	Uncertain significance	Uncertain significance	0.0000070	0.0000081
	COL5A1	9:134814873-C>G	NM_000093.4:c.3983C>G	NP_000084.3:p.Pro1328Arg	missense_variant	Benign	Likely benign(4);Uncertain significance(3)	B/LB	0.0001814	0.0004901
40	FBN1	15:048495155-G>A	NM_000138.4:c.2645C>T	NP_000129.3:p.Ala882Val	missense_variant	Pathogenic	Pathogenic/Likely pathogenic	P/LP	0	0
41	FBN1	15:048508633-A>G	NM_000138.4:c.1786T>C	NP_000129.3:p.Cys596Arg	missense_variant	Pathogenic	No record	No record	0	0

42	COL5A2	2:189053905-C>T	NM_000393.5:c.2489G>A	NP_000384.2:p.Arg830Gln	missense_variant	Likely Benign	No record	No record	0	0.0000244
	COL5A1	9:134766461-C>T	NM_000093.4:c.2096C>T	NP_000084.3:p.Thr699Met	missense_variant	Likely Benign	Likely benign(6);Uncertain significance(1)	B/LB	0.0005722	0.0006179
43	MYH11	16:015715004-G>C	NM_002474.3:c.5691C>G	NP_002465.1:p.Asn1897Lys	missense_variant	Likely Benign	No record	No record	0	0
	NOTCH1	9:136513479-CATT>C	NM_017617.5:c.2263_2265del	NP_060087.3:p.Asn755del	inframe_deletion	Benign	Uncertain significance	Uncertain significance	0.0002163	0.0002159
44	FBN1	15:048474645-TG>T	NM_000138.4:c.3969del	NP_000129.3:p.Asn1324MetfsTer89	frameshift_variant	Likely Pathogenic	No record	No record	0	0
	COL5A1	9:134699972-C>A	NM_000093.4:c.341C>A	NP_000084.3:p.Ala114Asp	missense_variant	Benign	Likely benign	B/LB	0.0007395	0.0005728
45	FBN1	15:048487353-G>A	NM_000138.4:c.3422C>T	NP_000129.3:p.Pro1141Leu	missense_variant	Benign	Benign(3);Likely benign(10);Uncertain significance(1)	B/LB	0.0005304	0.0008893
46	COL5A2	2:189062866-G>A	NM_000393.5:c.1976C>T	NP_000384.2:p.Pro659Leu	missense_variant	Benign	Likely benign(3);Uncertain significance(2)	B/LB	0.0003638	0.0004843
47	FBN1	15:048526254-AT>A	NM_000138.4:c.863del	NP_000129.3:p.Asp288ValfsTer42	frameshift_variant	Likely Pathogenic	No record	No record	0	0
	HCN4	15:073322516-C>G	NM_005477.3:c.3577G>C	NP_005468.1:p.Glu1193Gln	missense_variant	Benign	Benign(1);Likely benign(2);Uncertain significance(1)	B/LB	0.0006698	0.0007351
48	COL5A1	9:134753892-G>A	ENST00000371817.8:c.1762G>A	ENSP00000360882.3:p.Val588Met	missense_variant	Likely Benign	No record	No record	0.0000140	0.0000041
	NOTCH1	9:136509030-G>A	NM_017617.5:c.3011C>T	NP_060087.3:p.Ser1004Leu	missense_variant	Benign	Likely benign	B/LB	0.0001535	0.0001782
49	COL5A1	9:134753892-G>A	NM_000093.4:c.1762G>A	NP_000084.3:p.Val588Met	missense_variant	Likely Benign	No record	No record	0.0000140	0.0000041
50	FBN1	15:048489983-C>T	NM_000138.4:c.2950G>A	NP_000129.3:p.Val984Ile	missense_variant	Likely Pathogenic	Likely benign(5);Uncertain significance(4)	B/LB	0.0000279	0.0000162
51	COL1A1	17:050191451-C>T	NM_000088.3:c.2167G>A	NP_000079.2:p.Ala723Thr	missense_variant	Benign	Benign(3);Likely benign(1);Uncertain significance(1)	B/LB	0.0000838	0.0004508
52	FBN1	15:048520715-CG>C	NM_000138.4:c.1090del	NP_000129.3:p.Arg364AspfsTer31	frameshift_variant	Pathogenic	Pathogenic	P/LP	0	0
53	NUP43	6:149745973-A>T	NM_198887.3:c.210T>A	NP_942590.1:p.Asp70Glu	missense_variant	Uncertain Significance	No record	No record	0	0
54	FBN1	15:048437803-C>T	NM_000138.4:c.6278G>A	NP_000129.3:p.Gly2093Glu	missense_variant	Likely Pathogenic	No record	No record	0	0
55	FBN2	5:128276106-G>C	NM_001999.4:c.7526C>G	NP_001990.2:p.Thr2509Ser	missense_variant	Uncertain Significance	No record	No record	0.0000070	0
	FLNA	X:154349750-C>T	NM_001110556.2:c.7451G>A	NP_001104026.1:p.Arg2484His	missense_variant	Likely Benign	No record	No record	0.0000188	0.0000056
56	FLNA	X:154348990-G>C	NM_001110556.2:c.7803C>G	NP_001104026.1:p.Cys2601Trp	missense_variant	Uncertain Significance	No record	No record	0	0
57	FBN2	5:128259681-T>C	NM_001999.4:c.8513A>G	NP_001990.2:p.Gln2838Arg	missense_variant	Uncertain Significance	Uncertain significance	Uncertain significance	0	0.0000122
58	MFAP5	12:008648056-C>G	NR_123734.1:n.860G>C		splice_region_variant	Likely Benign	No record	No record	0.0000908	0.0000672
	COL1A1	17:050185845-T>C	ENST00000225964.9:c.4181A>G		missense_variant	Benign	Benign/Likely benign	B/LB	0.0008519	0.0007843
59	ACTA2	10:088948852-C>T	NM_001320855.1:c.79G>A	NP_001307784.1:p.Asp27Asn	missense_variant	Uncertain Significance	No record	No record	0	0
60	NOTCH1	9:136515476-C>T	NM_017617.5:c.1903+7G>A		splice_region_variant	Benign	No record	No record	0.0001605	0.0001039
61	MYH11	16:015750231-C>A	NM_022844.2:c.1965G>T	NP_074035.1:p.Gln655His	missense_variant	Uncertain Significance	No record	No record	0	0
	TGFBR2	3:030672168-G>A	ENST00000359013.4:c.1060G>A	ENSP00000351905.4:p.Ala354Thr	missense_variant	Uncertain Significance	Likely benign(2);Uncertain significance(3)	Uncertain significance	0.0000558	0.0000977
62	FBN1	15:048465600-TAGCCGGTTGGAC>T	NM_000138.4:c.4898_4909del	NP_000129.3:p.Cys1633_Gly1636del	inframe_deletion	Likely Pathogenic	No record	No record	0	0
	FBN2	5:128259727-G>T	NM_001999.4:c.8467C>A	NP_001990.2:p.Pro2823Thr	missense_variant	Uncertain Significance	No record	No record	0.0000070	0.0000041
63	MYH11	16:015756336-G>A	NM_022844.2:c.1749+5C>T		splice_region_variant	Uncertain Significance	No record	No record	0	0.0000041

64	FLNA	X:154354612-G>A	NM_001110556.2:c.5313+4C>T		splice_region_variant	Benign	Benign(2);Likely benign(1);Uncertain significance(1)	B/LB	0.0002712	0.0002383
65	ELN	7:074063360-G>A	ENST00000358929.8:c.2095G>A	ENSP00000351807.5:p.Ala699Thr	missense_variant	Likely Benign	Likely benign(1);Uncertain significance(2)	Uncertain significance	0.0000698	0.0000973
66	HCN4	15:073322516-C>G	NM_005477.3:c.3577G>C	NP_005468.1:p.Glu1193Gln	missense_variant	Benign	Benign(1);Likely benign(2);Uncertain significance(1)	B/LB	0.0006698	0.0007351
67	FBN1	15:048430707-C>T	NM_000138.4:c.6835G>A	NP_000129.3:p.Gly2279Arg	missense_variant	Likely Pathogenic	Uncertain significance	Uncertain significance	0.0000070	0.0000122
	FBN1	15:048505106-G>A	NM_000138.4:c.1879C>T	NP_000129.3:p.Arg627Cys	missense_variant	Pathogenic	Pathogenic	P/LP	0.0000070	0
68	FBN1	15:048427768-G>A	NM_000138.4:c.7003C>T	NP_000129.3:p.Arg2335Trp	missense_variant	Likely Pathogenic	Likely pathogenic(2);Uncertain significance(1)	P/LP	0	0
69	FBN1	15:048456720-C>T	NM_000138.4:c.5339G>A	NP_000129.3:p.Gly1780Glu	missense_variant	Likely Pathogenic	No record	No record	0	0
	HCN4	15:073322516-C>G	NM_005477.3:c.3577G>C	NP_005468.1:p.Glu1193Gln	missense_variant	Benign	enign(1);Likely benign(2);Uncertain significance(1)	B/LB	0.0006698	0.0007351
70	ACTA2	10:088939508-G>A	NM_001320855.1:c.807C>T	NP_001307784.1:p.Ile269%3D	splice_region_variant	Benign	Likely benign	B/LB	0.0000977	0.0001303
71	ELN	7:074063148-C>A	ENST00000358929.8:c.1973-5C>A		splice_region_variant	Uncertain Significance	No record	No record	0	0.0000088
72	ACTA2	10:088941252-C>T	NM_001320855.1:c.593G>A	NP_001307784.1:p.Arg198His	missense_variant	Uncertain Significance	Likely pathogenic(2);Uncertain significance(1)	P/LP	0	0
	SMAD3	15:067184779-CAG>C	NM_005902.4:c.925_926del	NP_005893.1:p.Ser309Ter	frameshift_variant	Likely Pathogenic	No record	No record	0	0
	HCN4	15:073322516-C>G	NM_005477.3:c.3577G>C	NP_005468.1:p.Glu1193Gln	missense_variant	Benign	Benign(1);Likely benign(2);Uncertain significance(1)	B/LB	0.0006698	0.0007351
	COL3A1	2:188990144-C>A	NM_000090.3:c.739C>A	NP_000081.1:p.Pro247Thr	missense_variant	Uncertain Significance	Uncertain significance	Uncertain significance	0	0
	COL5A1	9:134824844-A>G	ENST00000371817.8:c.4943A>G	ENSP00000360882.3:p.Asp1648Gly	missense_variant	Likely Benign	No record	No record	0.0000070	0.0000336
73	FBN1	15:048492577-T>G	NM_000138.4:c.2738A>C	NP_000129.3:p.Glu913Ala	missense_variant	Likely Pathogenic	Likely pathogenic	P/LP	0	0

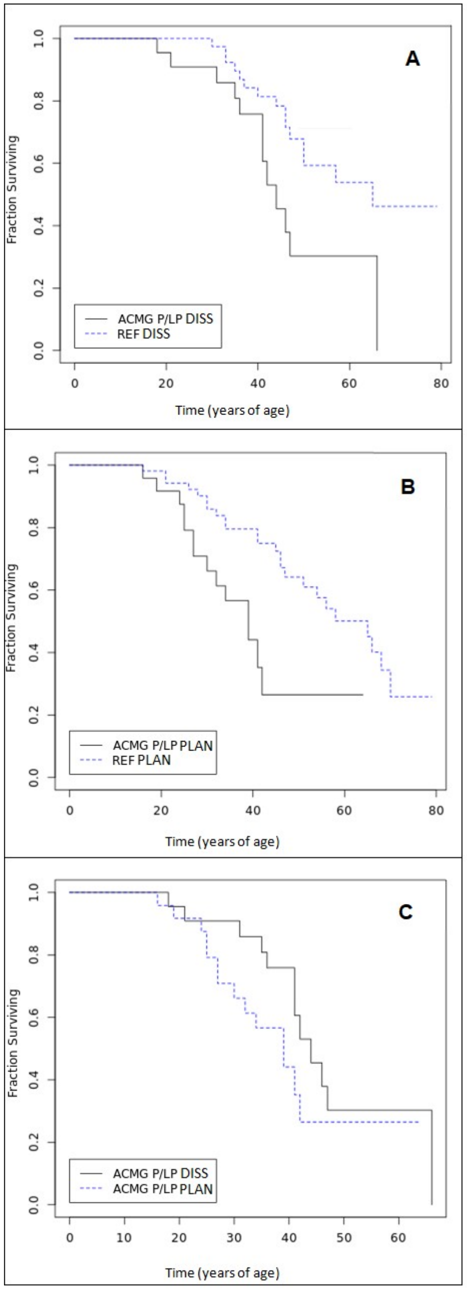

Figure S1. Kaplan–Meier analysis of event free survival in TAAD probands with variants classified as: (A) P/LP by ACMG (ACMG P/LP DISS) vs. reference group (REF DISS) – event = dissection prior to surgical intervention, Log-Rank Chi- square 5.23, p = 0.022), (B) P/LP by ACMG (ACMG P/LP PLAN) vs. reference group (REF PLAN) – event = planned prophylactic surgery, Log-Rank Chi- square 7.44, p = 0.0064, (C) P/LP by ACMG (ACMG P/LP DISS) – event = dissection prior to surgical intervention vs. P/LP by ACMG (ACMG P/LP PLAN) - event = planned prophylactic surgery, Log-Rank Chi-square 1.83, p = 0.18.

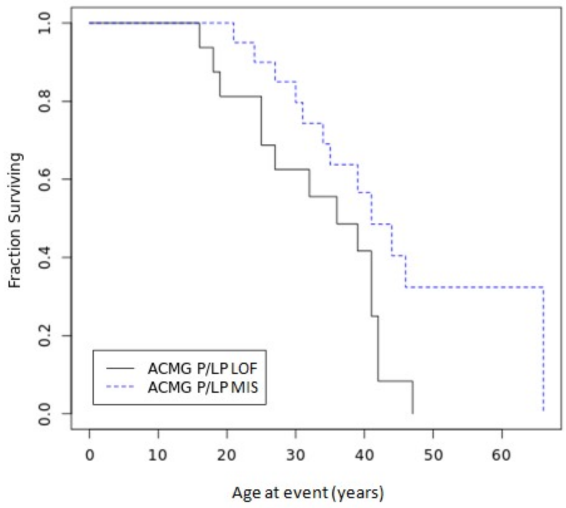

Figure S2. Kaplan–Meier analysis of event free survival in TAAD probands with LOF variants classified as P/LP by ACMG (ACMG P/LP LOF) vs. missense and small in-frame deletions (ACMG P/LP MIS), Log-Rank Chi-square 4.16, p = 0.041.
